# Association of Preferences for Participation in Decision-making With Care Satisfaction Among Hospitalized Patients

**DOI:** 10.1001/jamanetworkopen.2020.18766

**Published:** 2020-10-02

**Authors:** Gregory W. Ruhnke, Hyo Jung Tak, David O. Meltzer

**Affiliations:** 1 Section of Hospital Medicine, Department of Medicine, The University of Chicago, Chicago, Illinois; 2 Department of Health Services Research & Administration, University of Nebraska Medical Center, Omaha; 3 Center for Health and the Social Sciences, Harris School of Public Policy, The University of Chicago, Chicago, Illinois

## Abstract

**Importance:**

The association of patient desire to participate in health care decisions with care satisfaction is poorly understood. The contributions of such desire, expectations of care, and quality of care in assessing patient satisfaction are not known.

**Objective:**

To investigate the association of hospitalized patients’ desire to delegate decisions to their physician with care dissatisfaction.

**Design, Setting, and Participants:**

Survey study in an academic research setting. As part of The University of Chicago Hospitalist Study, data were collected on 13 902 hospitalized patients admitted to the general internal medicine service of The University of Chicago Medical Center between July 1, 2004, and September 30, 2012, who answered an inpatient survey administered soon after the time of admission and a 30-day follow-up survey. The dates of analysis were January 2014 to June 2015.

**Exposure:**

Patient-reported preference to leave medical decisions to their physician (definitely agree or somewhat agree vs somewhat disagree or definitely disagree).

**Main Outcomes and Measures:**

The main outcomes were patient-reported dissatisfaction with overall service, dissatisfaction with physician care, and lack of confidence and trust in the physicians providing treatment, which were obtained from the 30-day follow-up survey.

**Results:**

The sample population included 13 902 patients (mean [SD] age, 56.7 [19.1] years; 60.4% female [n = 8397] and 74.2% African American [n = 10 310]) who completed both surveys. Overall, 53.2% had no higher educational attainment, 22.7% were insured by Medicaid, and 51.1% reported a general self-assessed health status of fair or poor. The proportions of respondents who agreed and disagreed with delegating decisions to the responsible physician were 71.1% and 28.9%, respectively. A statistically significantly higher proportion of those who agreed rated their overall care as excellent or very good compared with those who disagreed (68.0% vs 62.5%; *P* < .001). Similarly, a statistically significantly higher proportion of those who agreed were extremely satisfied with the physician care received (67.8% vs 62.5%; *P* < .001). In the multivariable logistic regression models, compared with those patients who definitely agreed with delegation, patients who definitely disagreed were more likely to be dissatisfied with overall service (odds ratio [OR], 1.86; 95% CI, 1.54-2.24) and the physician care received (OR, 1.78; 95% CI, 1.42-2.22) and lack confidence and trust in the physicians providing treatment (OR, 2.05; 95% CI, 1.62-2.59).

**Conclusions and Relevance:**

The findings suggest that patient preferences to participate in medical decision-making are statistically significantly associated with dissatisfaction of hospitalized patients. Clinicians should individualize their encouragement of patient participation in diagnostic and management decisions to maximize patient satisfaction.

## Key Points

**Question:**

Are patient preferences for participation in medical decisions associated with measures of satisfaction?

**Findings:**

In this survey study of 13 902 hospitalized patients, a statistically significantly greater proportion of patients who preferred to delegate decisions to their physician were satisfied with their care compared with patients who preferred not to delegate. Adjusting for observed patient characteristics, those with a nondelegating preference were more dissatisfied with overall service and physician care and lacked confidence and trust in the physicians providing treatment.

**Meaning:**

The findings of this study suggest that expectations of care and communication that accompany a desire to participate in decisions may be associated with patient dissatisfaction or distrust.

## Teaser

This survey study investigates the association of hospitalized patients’ desire to delegate decisions to their physician with care dissatisfaction.

## Introduction

Greater patient participation in medical decisions improves satisfaction^1^ and objective health outcomes^2^ while fostering clinical care more strongly aligned with patient preferences.^3^ Accordingly, numerous initiatives encourage patients to actively participate in decisions, including the Affordable Care Act.^4^

Patient preference for engagement in medical decision-making varies across individuals, subgroups, and decision types. Patients generally want to receive information^5^ but often wish to delegate decisions to physicians,^6,7,8,9^ a preference that may be stronger among older patients.^8,10^ Patient preferences for engagement in decisions may differ based on technical components of the decision and subjective aspects of the outcomes.^1,5^

Variation in patient preferences and expectations concerning engagement may be associated with patient-reported quality metrics, including satisfaction,^11^ ratings of care,^12^ quality of life,^13^ and other measures of health service quality.^14^ Because preferences and expectations exhibit geographic variation,^15^ such associations could complicate the interpretation of patient-reported outcomes (PROs) as measures of clinician performance. Despite the growing use of such performance metrics,^16^ literature on the association of PROs with preferences for participation in decision-making is limited, particularly among urban minority racial/ethnic populations. Because race/ethnicity has important associations with patient trust^17^ and satisfaction,^18^ studies that include diverse populations are essential to understand preferences among diverse groups and their implications for PROs.

Prior research regarding the consequences of patients’ desire to participate in health care decisions has been limited by small sample sizes, lack of adjustment for important confounding factors, and selection bias resulting from restriction to educated populations with sufficient ability to answer questionnaires.^19,20^ We studied the association of preferences for participation in clinical decisions with care satisfaction among a predominantly minority racial/ethnic sample of hospitalized patients at The University of Chicago Medical Center, an urban academic medical center.

## Methods

### Data Sources and Study Population

This survey study in an academic research setting used data collected through the University of Chicago Hospitalist Study,^21^ which merges administrative and patient survey data for hospitalized general medicine patients. Data were collected on 13 902 hospitalized patients admitted to the general internal medicine service of The University of Chicago Medical Center between July 1, 2004, and September 30, 2012, who answered an inpatient survey administered soon after the time of admission and a 30-day follow-up survey. The dates of analysis were January 2014 to June 2015. The study was approved by The University of Chicago Medical Center Institutional Review Board and followed the Strengthening the Reporting of Observational Studies in Epidemiology (STROBE) reporting guideline. Trained research assistants obtained written informed consent from all admitted patients immediately after admission for an inpatient survey with 44 questions, including race/ethnicity, educational attainment, general self-assessed health status, and patient preference for medical decision-making. We contacted patients 30 days after discharge to ask 56 follow-up questions, including postdischarge medical care use, health status, and service quality measures during the previous hospitalization.

### Data Elements

The following 3 service quality measures were obtained from the 30-day follow-up survey: overall rating of care received (excellent, very good, good, fair, or poor), satisfaction with physician care (extremely satisfied, somewhat satisfied, somewhat dissatisfied, or extremely dissatisfied), and confidence and trust in the physicians providing treatment (yes always, yes sometimes, or no). Poor service quality measures were defined as follows: dissatisfaction with overall care (fair or poor), physician care (extremely or somewhat dissatisfied), and no confidence and trust (vs yes always or sometimes).

The primary independent variable was patient-reported preference to leave medical decisions to their physician derived from a survey item (“I prefer to leave decisions about my medical care up to my doctor”) to which patients responded as definitely agree or somewhat agree vs somewhat disagree or definitely disagree. Additional detail regarding this variable has been previously published.^8,9^ Other patient-level independent variables include the following: age, sex, race/ethnicity, educational attainment (any high school or less, high school graduate, some college or junior college, college graduate, or any graduate-level educational attainment), insurance type (private, Medicare, Medicaid, or no insurance), general self-assessed health status (excellent, very good, good, fair, or poor), Charlson Comorbidity Index score, and transfer from another health care facility or service. The main outcomes were patient-reported dissatisfaction with overall service, dissatisfaction with physician care, and lack of confidence and trust in the physicians providing treatment, which were obtained from the 30-day follow-up survey.

### Statistical Analysis

For the bivariate analyses, we used *t* test (age) and Pearson χ^2^ test to examine the differences in patient characteristics and service quality measures with statistical significance prespecified at *P* < .05. To investigate the association of patient preference for medical decision-making with the 3 service quality measures, we specified multivariable logistic regression models, with the 4-level preference indicator (definitely agree or somewhat agree vs somewhat disagree or definitely disagree) as the primary independent variable (with definitely agree as the reference category). The independent covariates were the patient-level variables described previously and categorical variables for year, month, attending physician, the 10 most frequent principal diagnoses, and admission on a weekend. The dependent variables of each regression were reverse-coded derived from the following service quality measures: dissatisfaction with overall service (rating of fair or poor vs excellent, very good, or good), dissatisfaction with physician care (somewhat dissatisfied or extremely dissatisfied), and lack of confidence and trust in the physicians providing treatment (vs yes always or yes sometimes).

A small amount of missing data (5.8% for educational attainment, 2.8% for insurance type, 5.0% for general self-assessed health status, 8.2% for patient preference for medical decision-making, 4.1% for overall rating of care received, 3.2% for satisfaction with physician care, and 3.7% for confidence and trust in the physicians providing treatment) was imputed using multiple imputation methods with 20 iterations, which yielded consistent estimates with valid inference in estimation.^22^ We used an ordered logit model for ordinal variables (educational attainment, general self-assessed health status, patient preference for medical decision-making, overall rating of care received, satisfaction with physician care, and confidence and trust in the physicians providing treatment) and a multinomial logit model for the nonordinal variable (insurance type). To confirm the robustness of the imputations, we compared the results with and without imputations. Statistical analyses were performed with Stata, version 11.2 (StataCorp LLC).

## Results

The sample population included 13 902 admissions for which both the initial inpatient and 30-day follow-up surveys were completed. Between July 1, 2004, and September 30, 2012, there were 35 682 patients admitted to the general medicine service at The University of Chicago Medical Center, of which 3228 were not surveyed because they completed the survey during an admission within the prior 60 days. Of the remaining 32 454 eligible hospitalized patients, 22 954 (70.7%) completed the inpatient survey. The others were discharged without completing the survey. Compared with participants who responded to the inpatient survey, nonparticipants were older, were more likely to be African American, were less healthy, and had a shorter length of stay and reduced total hospitalization cost. Among the inpatient survey participants, 13 902 of 22 954 patients (60.6%) completed the 30-day follow-up survey. For the 30-day follow-up survey, nonparticipants were more likely than participants to be male, less educated, and less healthy and were more likely to be publicly insured or uninsured.

Table 1 lists patient sociodemographic characteristics, general self-assessed health status, and patient preference for medical decision-making. The mean (SD) patient age was 56.7 (19.1) years, 60.4% (n = 8397) were women, 39.6% (n = 5505) were men, and 74.2% were African American. Overall, 53.2% had no higher educational attainment, 22.7% were insured by Medicaid, and 51.1% reported a general self-assessed health status of fair or poor. The proportions of respondents who agreed and disagreed with delegating decisions to the responsible physician were 71.1% and 28.9%, respectively. A statistically significantly higher proportion of those who agreed rated their overall care as excellent or very good compared with those who disagreed (68.0% vs 62.5%; *P* < .001). Similarly, a statistically significantly higher proportion of those who agreed were extremely satisfied with the physician care received (67.8% vs 62.5%; *P* < .001).

**Table 1.** Patient and Hospitalization Characteristics, Preferences for Participation in Medical Decisions, and Service Quality Measures

Variable	No. (%)			*P* value
	Total study population (N = 13 902)	Preference for delegating decisions to their doctor		
		Agree (n = 9878)	Disagree (n = 4024)	
**Patient characteristics and general self-assessed health status**				
Age, mean (SD), y	56.7 (19.1)	57.6 (19.2)	54.7 (18.6)	<.001
Female sex	8397 (60.4)	5860 (59.3)	2537 (63.0)	<.001
African American race	10 310 (74.2)	7433 (75.2)	2877 (71.5)	<.001
Educational attainment				
Any high school or less	3003 (21.6)	2350 (23.8)	653 (16.2)	<.001
High school graduate	4391 (31.6)	3275 (33.2)	1116 (27.7)	
Some college or junior college	3809 (27.4)	2596 (26.3)	1213 (30.1)	
College graduate	1636 (11.8)	1042 (10.5)	594 (14.8)	
Any graduate-level educational attainment	1063 (7.6)	619 (6.3)	444 (11.0)	
Insurance type				
Private	3129 (22.5)	2051 (20.8)	1078 (26.8)	<.001
Medicare	7032 (50.6)	5156 (52.2)	1876 (46.6)	
Medicaid	3160 (22.7)	2267 (22.9)	893 (22.2)	
No insurance	481 (3.5)	340 (3.4)	141 (3.5)	
General self-assessed health status				
Excellent	1089 (7.8)	772 (7.8)	317 (7.9)	<.001
Very good	1625 (11.7)	1182 (12.0)	443 (11.0)	
Good	4085 (29.4)	2905 (29.4)	1180 (29.3)	
Fair	4396 (31.6)	3130 (31.7)	1266 (31.5)	
Poor	2707 (19.5)	1893 (19.2)	814 (20.2)	
Charlson Comorbidity Index score, mean (SD)	1.7 (1.7)	1.7 (1.7)	1.7 (1.7)	.36
10 Most frequent principal diagnoses				
Complications associated with procedures (eg, catheters)	675 (4.9)	475 (4.8)	200 (5.2)	.69
Diabetes	619 (4.5)	440 (4.5)	179 (4.4)	.99
Asthma	537 (3.9)	393 (4.0)	144 (3.6)	.27
Acute kidney failure	523 (3.8)	372 (3.8)	151 (3.8)	.97
Sickle cell anemia	475 (3.4)	294 (3.0)	181 (4.5)	<.001
Pneumonia, organism unspecified	429 (3.1)	303 (3.1)	126 (3.1)	.84
Cellulitis and abscess	399 (2.9)	280 (2.8)	119 (3.0)	.69
Pancreatitis and pancreatic pseudocyst	329 (2.4)	235 (2.4)	94 (2.3)	.88
Disorders of fluids, electrolytes, and acid-base balance	312 (2.2)	223 (2.3)	89 (2.2)	.87
Gastrointestinal hemorrhage	311 (2.2)	227 (2.3)	84 (2.1)	.45
Transfer from another health care facility or service	1396 (10.0)	989 (10.0)	407 (10.1)	.86
Admission on a weekend	3433 (24.7)	2468 (25.0)	965 (24.0)	.23
Patient preference for medical decision-making: “I prefer to leave decisions about my medical care up to my doctor”				
Definitely agree	5247 (37.7)	5247 (53.1)	NA	NA
Somewhat agree	4631 (33.3)	4631 (46.9)	NA	NA
Somewhat disagree	2230 (16.0)	NA	2230 (55.4)	NA
Definitely disagree	1794 (12.9)	NA	1794 (44.6)	NA
**3 Service quality measures**				
Overall rating of care received				
Excellent	5368 (38.6)	3974 (40.2)	1394 (34.6)	<.001
Very good	3873 (27.9)	2751 (27.8)	1122 (27.9)	
Good	3328 (23.9)	2319 (23.5)	1009 (25.1)	
Fair	948 (6.8)	606 (6.1)	342 (8.5)	
Poor	385 (2.8)	228 (2.3)	157 (3.9)	
Satisfaction with physician care				
Extremely satisfied	9212 (66.3)	6698 (67.8)	2514 (62.5)	<.001
Somewhat satisfied	3788 (27.2)	2623 (26.6)	1165 (29.0)	
Somewhat dissatisfied	540 (3.9)	334 (3.4)	206 (5.1)	
Extremely dissatisfied	362 (2.6)	223 (2.3)	139 (3.5)	
Confidence and trust in the physicians providing treatment				
Yes always	10 965 (78.9)	7985 (80.8)	2980 (74.1)	<.001
Yes sometimes	2261 (16.3)	1494 (15.1)	767 (19.1)	
No	676 (4.9)	399 (4.0)	277 (6.9)	

Abbreviation: NA, not applicable.

**Figure 1.**
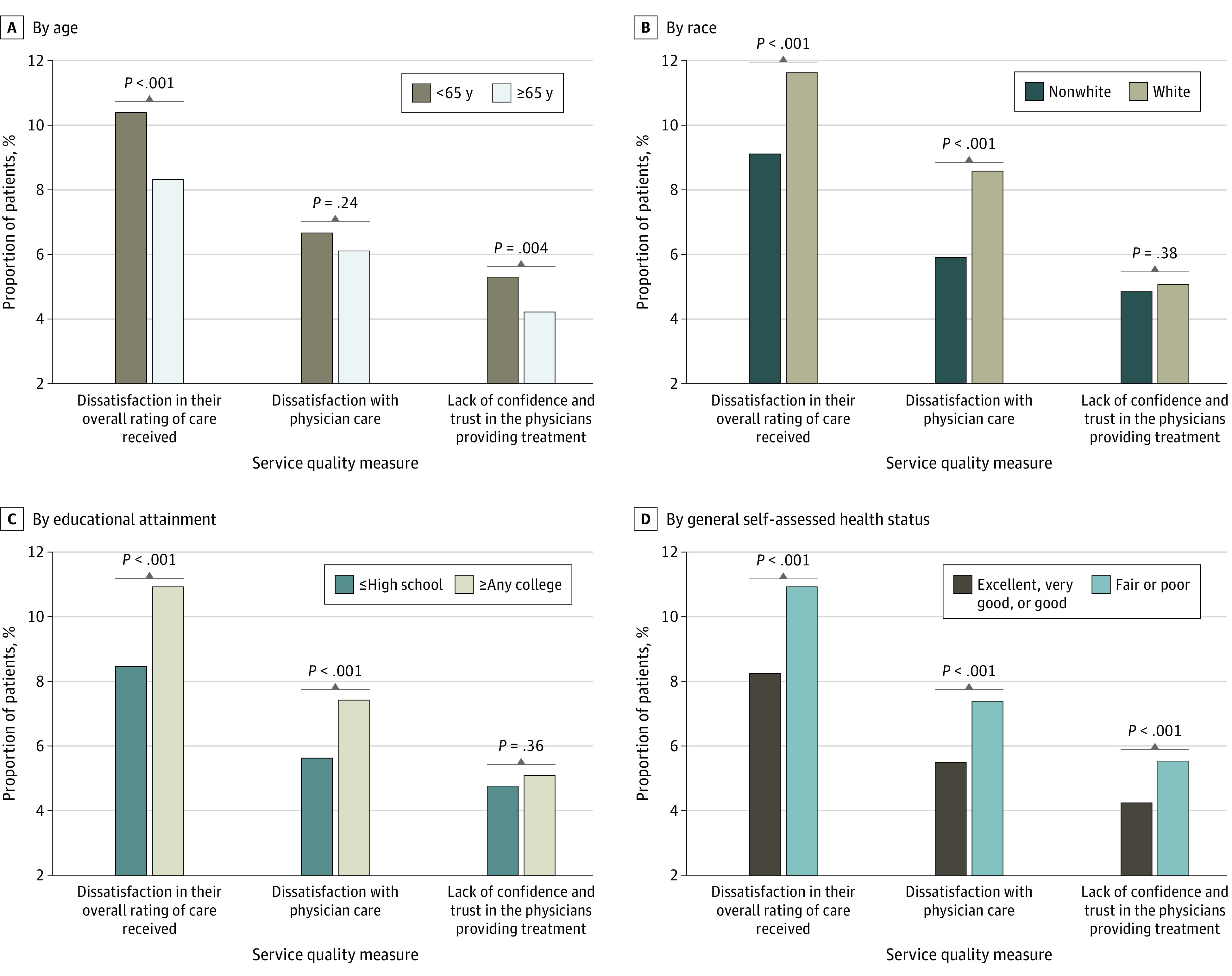
Service Quality Measures Stratified by Baseline Characteristics A-D, The bars indicate the proportion of respondents by specified category who were dissatisfied with overall care received, dissatisfied with physician care received, and lacked confidence and trust in the physicians providing treatment.

Table 1 also lists the responses to the 3 service quality measures: 9.6% evaluated the overall rating of care received as fair or poor, 6.5% expressed dissatisfaction with physician care, and 4.9% did not have confidence and trust in the physicians providing treatment. Figure 1 shows that the characteristics of age younger than 65 years, White race, higher educational attainment, and worse general self-assessed health status were associated with greater degrees of dissatisfaction in the overall rating of care received. White race, higher educational attainment, and worse general self-assessed health status were also associated with greater degrees of dissatisfaction with physician care. The characteristics of age younger than 65 years and worse general self-assessed health status were associated with greater lack of confidence and trust in the physicians providing treatment.

**Figure 2.**
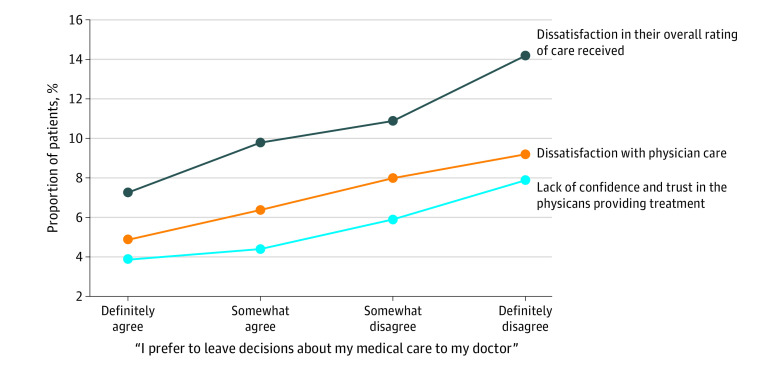
Proportion of Patients Giving Unfavorable Ratings on the 3 Service Quality Measures Reduced willingness to defer medical decisions to the treating physician was associated with greater dissatisfaction in overall rating of care received, greater dissatisfaction with physician care, and reduced confidence and trust in the treating physicians.

Figure 2 shows the association of patient preference for medical decision-making with patient-reported measures of dissatisfaction and lack of confidence and trust in the physicians providing treatment. Reduced willingness to defer medical decisions to the physicians providing treatment was associated with greater dissatisfaction in the overall care received, more dissatisfaction with physician care, and reduced confidence and trust in the physicians providing treatment. Compared with patients who definitely preferred to leave medical decisions to their physician, those who definitely disagreed with delegating decisions to their physician were more likely to be dissatisfied in the overall care received (14.2% vs 7.3%), express dissatisfaction with physician care (9.2% vs 4.9%), and lack confidence and trust in the physicians providing treatment (7.9% vs 3.9%) (*P* < .001 for all).

**Figure 3.**
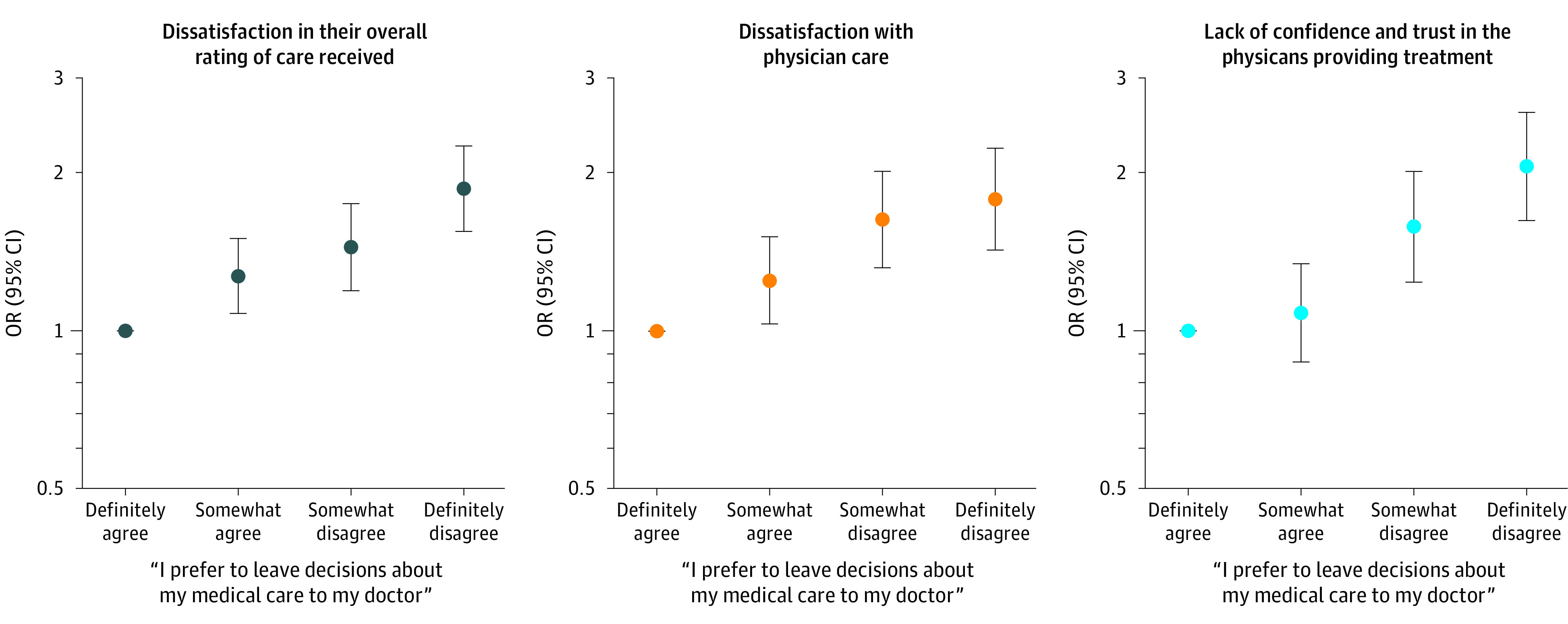
Association of Patient Preferences for Participation in Decision-making With Care Satisfaction OR indicates odds ratio.

Figure 3 shows the multivariable-adjusted association of patient preference for medical decision-making with the 3 service quality measures. Table 2 lists the full estimation results. Compared with patients who strongly preferred to leave medical decisions to their physician, those who definitely disagreed with delegating decisions were more likely to be dissatisfied in the overall care received (odds ratio [OR], 1.86; 95% CI, 1.54-2.24), express dissatisfaction with physician care (OR, 1.78; 95% CI, 1.42-2.22), and lack confidence and trust in the physicians providing treatment (OR, 2.05; 95% CI, 1.62-2.59). The findings were unchanged when the analysis was restricted to patients without missing data (ie, without imputations).

**Table 2.** Association of Patient Preferences for Participation in Medical Decisions With Care Satisfaction Across the 3 Service Quality Measures

Variable	OR (95% CI)^a^		
	Dissatisfaction with overall care received (N = 13 902)	Dissatisfaction with physician care (N = 13 902)	Lack of confidence and trust in the physicians providing treatment (N = 13 902)
Centered age	0.99 (0.99-1.00)	1.00 (0.99-1.00)	0.99 (0.98-1.00)
Centered age squared	1.00 (1.00-1.00)	1.00 (1.00-1.00)	1.00 (1.00-1.00)
Female sex	1.08 (0.95-1.22)	1.05 (0.91-1.22)	1.03 (0.87-1.22)
African American race	0.82 (0.71-0.95)	0.80 (0.68-0.95)	0.89 (0.72-1.09)
Educational attainment			
Any high school or less	1 [Reference]	1 [Reference]	1 [Reference]
High school graduate	1.10 (0.91-1.33)	1.01 (0.80-1.26)	0.85 (0.67-1.08)
Some college or junior college	1.28 (1.06-1.56)	1.08 (0.86-1.35)	0.91 (0.71-1.16)
College graduate	1.45 (1.16-1.81)	1.44 (1.10-1.87)	0.87 (0.63-1.20)
Any graduate-level educational attainment	1.39 (1.07-1.80)	1.48 (1.11-1.97)	0.98 (0.69-1.40)
Insurance type			
Private	1 [Reference]	1 [Reference]	1 [Reference]
Medicare	1.24 (1.04-1.49)	1.13 (0.92-1.40)	1.32 (1.02-1.69)
Medicaid	1.20 (0.99-1.44)	0.87 (0.69-1.10)	1.18 (0.91-1.54)
No insurance	0.76 (0.51-1.15)	0.71 (0.43-1.16)	1.01 (0.61-1.67)
General self-assessed health status			
Excellent	1 [Reference]	1 [Reference]	1 [Reference]
Very good	0.90 (0.66-1.21)	0.88 (0.61-1.25)	0.90 (0.60-1.36)
Good	1.05 (0.81-1.36)	0.94 (0.69-1.28)	0.94 (0.66-1.34)
Fair	1.28 (0.99-1.65)	1.15 (0.84-1.56)	1.19 (0.85-1.68)
Poor	1.56 (1.20-2.03)	1.48 (1.08-2.03)	1.36 (0.94-1.95)
Charlson Comorbidity Index score	1.01 (0.97-1.04)	1.03 (0.99-1.08)	1.01 (0.96-1.06)
10 Most frequent principal diagnoses			
All other	1 [Reference]	1 [Reference]	1 [Reference]
Complications associated with procedures (eg, catheters)	1.11 (0.85-1.45)	0.87 (0.62-1.23)	0.83 (0.57-1.22)
Diabetes	0.95 (0.70-1.28)	0.95 (0.65-1.38)	0.98 (0.66-1.45)
Asthma	0.64 (0.44-0.93)	0.71 (0.46-1.11)	0.46 (0.26-0.80)
Acute kidney failure	0.80 (0.56-1.13)	0.86 (0.58-1.27)	0.76 (0.47-1.23)
Sickle cell anemia	1.50 (1.12-2.01)	1.73 (1.21-2.49)	1.28 (0.87-1.89)
Pneumonia, organism unspecified	0.78 (0.53-1.15)	1.01 (0.67-1.51)	1.03 (0.64-1.64)
Cellulitis and abscess	1.32 (0.95-1.83)	1.17 (0.78-1.76)	0.92 (0.55-1.54)
Pancreatitis and pancreatic pseudocyst	0.72 (0.47-1.11)	0.83 (0.51-1.34)	0.65 (0.35-1.21)
Disorders of fluids, electrolytes, and acid-base balance	1.28 (0.88-1.86)	1.09 (0.68-1.73)	1.06 (0.63-1.78)
Gastrointestinal hemorrhage	0.93 (0.61-1.42)	0.61 (0.34-1.10)	0.81 (0.44-1.48)
Transfer from another health care facility or service	0.96 (0.78-1.17)	1.13 (0.90-1.42)	1.00 (0.76-1.31)
Admission on a weekend	0.87 (0.76-1.01)	1.06 (0.90-1.25)	1.15 (0.96-1.37)
Patient preference for medical decision-making: “I prefer to leave decisions about my medical care up to my doctor”			
Definitely agree	1 [Reference]	1 [Reference]	1 [Reference]
Somewhat agree	1.27 (1.08-1.50)	1.25 (1.03-1.50)	1.08 (0.87-1.34)
Somewhat disagree	1.44 (1.19-1.74)	1.62 (1.31-2.01)	1.57 (1.24-2.00)
Definitely disagree	1.86 (1.54-2.24)	1.78 (1.42-2.22)	2.05 (1.62-2.59)

Abbreviation: OR, odds ratio.

^a^ Odds ratios and 95% CIs were adjusted for all independent variables in multivariable logistic regression models.

## Discussion

The core elements of shared decision-making are physician sharing of information and patient participation in decisions, which may improve patient satisfaction and health outcomes.^23^ However, in the present study, a desire to participate in decisions was associated with reduced satisfaction and less confidence and trust in the physicians providing treatment. Prior literature has found that patient preference for involvement in decisions is heterogeneous^7,19^ and is inconsistently associated with outcomes^24^ and that physicians may overestimate patients’ desire to make decisions.^25^ Given this finding and the increasing use of PROs in performance reporting, understanding the implications of patients having a larger role in decisions for PROs is critical. Although we cannot know with certainty the reasons for the observed association between a greater desire to participate in decisions and reduced satisfaction and trust, we hypothesize that an increased desire to participate in decision-making is associated with greater expectations of care and communication.^26^ However, alternative possibilities not mediated directly by expectations should be recognized. For example, prior suboptimal clinical interactions may concurrently impact both patients’ preferences for participation and evaluations of care temporally proximate to survey completion.

If the associations that were observed in the present study were indeed mediated by expectations, the burdens of decision-making for patients desiring active participation or the occurrence of insufficient communication (relative to expectations) may result in the observed dissatisfaction. Because preferences for participation in decisions were asked of patients early in their hospitalization, these are unlikely to result from care received during that hospitalization but rather relate to patient characteristics or prior health care experiences.^27^ This study’s importance also lies in its urban, largely African American sample of hospitalized patients with little higher educational attainment. Understanding the association of preferences with satisfaction is important in this understudied group because of persistent disparities in care and satisfaction, especially because prior literature suggests racial/ethnic differences in preferences for participation in medical decisions.^7^

The medical community’s desire for greater patient participation in decisions arises from a theory based on sociopolitical factors,^24^ including cultural predispositions for patient autonomy^6,28^ and changing views regarding the ethical aspects of decision-making.^1^ However, there is some evidence for greater satisfaction and improved outcomes as a result of patient participation in decisions.^19,20^ Although definitive research in this area is limited, the discrepancy between prior literature and the present study may be worth exploring. First, much literature has studied healthy volunteers or outpatients.^3^ Based on a sample of hospitalized patients, our findings may reflect a vulnerability that modifies the consequences of preferences for participation on care satisfaction and trust.

Second, many previous studies on this subject have been based on middle-class or upper-class predominantly White populations,^3,18,32^ in contrast to this urban African American sample. Preferences for care and decision-making differ markedly across patient populations and over time.^7^ These considerations may explain in part why we did not observe greater distrust among the African American patients in this study, as other studies have.^29^ Furthermore, distrust is associated with receipt of care inconsistent with strongly held patient preferences and with lack of patient centeredness.^29^ Because vulnerable patients like those in this sample with a preference for physician-directed decision-making are less likely to have solidified, immutable expectations of care, the absence of conflict between preferences and values may have assuaged the degree of distrust.

Third, several previous relevant studies^27,30,31^ were based on interventions directed at improving satisfaction through shared decision-making. This study differs in that it evaluates the associations between preexisting patient desire to participate in decisions, patient motivation for participation, and satisfaction. We cannot know with certainty the contributions of innate patient characteristics and prior health care experiences in generating patient desire to participate in decisions. However, we believe that greater expectations of care and communication among patients who do not want to delegate decision-making may create increased potential for dissatisfaction and lack of trust. The consistency of associations found across 2 measures (satisfaction with overall service and physician care) of satisfaction and confidence and trust suggests that the connections between patient preferences and outcomes are meaningful. Greater expectations may both give rise to a desire to participate in decisions and create more opportunity for dissatisfaction.^12^ We cannot know with certainty the mechanism of the associations found in this study; however, based on theory and previous literature,^32,33^ we believe that higher expectations of care and communication that arise among assertive patients with a greater desire to participate in decision-making mediate the findings that we observed.

### Implications for Future Research

There are several important implications of this study. The associations we found suggest that efforts to involve patients in shared decision-making should be sensitive to the heterogeneity of patients’ preferences to participate and encourage only types of involvement concordant with their wishes. This implication supports concerns in the published literature suggesting caution regarding overzealous efforts to involve patients because many do not wish to have active participation.^7^ Efforts to involve patients in health care decisions should be individualized and reflect the importance of shared decision-making in maximizing satisfaction for a substantial proportion of patients. However, our subgroup analyses in Figure 1 suggest that clinicians should remain cognizant of the fact that age, race/ethnicity, educational attainment, and general self-assessed health status could have important implications for how patient involvement may alter their satisfaction, perceptions of trust, and confidence in the physicians providing treatment. The findings of this study suggest that patient desire to participate in decisions would be appropriate to include in surveys (eg, the Hospital Consumer Assessment of Healthcare Providers and Systems^34^), especially because hospital rankings vary substantially by patient characteristics.

Furthermore, given the geographic variation in patient preferences,^15^ the mix of patients at a given hospital may have consequences for PROs.^35^ Although we cannot know with certainty if the associations that we observed would also exist in patient populations with alternative racial/ethnic and educational attainment distributions, this study describes important implications in an understudied population that may offer insights applicable across broad populations. Although many national policies, including the Affordable Care Act,^4^ encourage shared decision-making, a substantial proportion of patients do not desire an active role in medical decisions.^7,25^ Institutions with patient populations less willing to defer decisions to physicians may be at risk for poor ratings on publicly reported measures of satisfaction. In addition to other recognized challenges of such reporting,^16^ this implication may create substantial difficulty in the interpretability of statistics based on patient surveys.

In addition, performance reporting may create a disincentive for hospitals to encourage an environment of shared decision-making. In this study, patients with fair or poor general self-assessed health status were consistently more dissatisfied than those with better health across the 3 service quality measures. In addition to the consequences that this association may have for PROs at institutions with a disproportionately large patient base with poor health, critical access hospitals and other hospitals with low operating margins tend to take care of sicker patients.^36^ Because poor ratings of satisfaction are associated with higher rates of emergency department use,^37^ patients in poor health who are also dissatisfied with care received may be large contributors to statistics on ambulatory-sensitive conditions.

### Limitations

This study has limitations. First, the results of this study at a single medical center may not be generalizable outside of the urban, largely African American population we studied. Second, 29.3% of admitted patients did not participate in the survey. However, although there were certain differences in baseline characteristics between participants and nonparticipants, the magnitude of these variations was small. Therefore, selection bias is unlikely to have impacted our primary findings.

Third, we studied hospitalized patients, for whom acute illness may modify satisfaction and confidence and trust. This is in contrast to the absence of acute illness in the outpatient setting, where physician-patient relationships are more durable and there is greater continuity of care. In addition, dissatisfaction may be associated with a lack of willingness to defer decisions. However, because patients were asked about their preference for participation soon after hospitalization, the temporal association suggests that these preferences would be based on prior health care experiences and innate patient characteristics, rather than dissatisfaction with care received after survey administration. Also, prior experience may alter patient satisfaction with current health care. However, satisfaction during a current or recent hospitalization is the strongest determinant of patient satisfaction with care.^32,33^

Fourth, although shared decision-making requires participation of both physicians and patients, we do not have information on physician preferences to engage in shared decision-making. However, because physicians on the general internal medicine service at The University of Chicago Medical Center are assigned to patients based on a fixed call schedule, the decision-making preferences of physicians should be similar across categories of patient preference for medical decision-making. The inclusion of attending physician in our multivariable logistic regression models also minimizes potential consequences of physician preferences, although we cannot know with certainty how physician behavior might differ according to physician-patient relationships.

Fifth, we have not measured actual participation (but rather preferences for such) or other contributing factors (eg, personality) and cannot draw definitive conclusions regarding the mechanism of the observed associations. Although we used delegation of decisions as a proxy measure of desire for shared decision-making, future research may evaluate how robust the observed associations are to alternative measures of shared decision-making.

Sixth, despite potentially important associations,^38^ we did not measure the consequences of participation in decision-making on objective health outcomes. If a preference for participation in medical decisions improves health outcomes, such benefits may outweigh the satisfaction-reducing associations we observed with PROs. Patient participation in decision-making may help physicians exercise responsible stewardship, which may reduce unnecessary resource use while maintaining or improving outcomes.

## Conclusions

This survey study provides insights regarding the heterogeneous association that patient preferences for participation in decisions and shared decision-making may have with patient satisfaction. Within certain patient populations, expectations of care and communication that accompany a desire to participate in health care decisions may deleteriously alter satisfaction. Clinicians should individualize their encouragement of patient participation, which can have consequences on satisfaction and trust. Finally, organizations engaged in public dissemination of PROs as quality measures should recognize the dependence of such outcomes on patient characteristics.
